# Radiological measurements of lacrimal gland in thyroid eye disease

**DOI:** 10.1007/s10792-024-02991-4

**Published:** 2024-02-06

**Authors:** Carmelo Caltabiano, Khizar Rana, Mark B. Beecher, Dinesh Selva

**Affiliations:** 1 Discipline of Ophthalmology and Visual Sciences, University of Adelaide, North Terrace, Adelaide, SA 5000 Australia; 2 South Australian Institute of Ophthalmology, Royal Adelaide Hospital, Port Road, Adelaide, SA 5000 Australia

**Keywords:** Thyroid eye disease, Lacrimal gland, Enlargement, Correlation, Dimensions, Volume

## Abstract

**Purpose:**

Lacrimal gland enlargement is a common feature of thyroid eye disease (TED) and has been positively correlated with the clinical activity score. Although lacrimal gland volume is the preferred measure of lacrimal gland size, its calculation is not clinically translatable due to the expertise, time and advanced software required. The aim of our study is to determine whether the lacrimal gland volume in patients with TED undergoing magnetic resonance imaging (MRI) can be estimated using simpler lacrimal gland linear and area measurements.

**Methods:**

A retrospective review of 102 orbits (51 patients) with TED who underwent orbital MRI was conducted. The maximum length, width, and area of the lacrimal gland were measured in axial and coronal sections. Lacrimal gland volume was calculated by using a manual segmentation technique on all consecutive axial slices on commercially available software, OsiriX. All quantitative measurements were correlated with the lacrimal gland volume.

**Results:**

Mean age of participants was 59 ± 16 years, and 67% (*n* = 34) were females. With multivariate analyses, combined lacrimal gland axial and coronal areas strongly correlated with volume (*r* = 0.843, *p* < 0.01). Strong univariate predictors of volume included axial area (*r* = 0.704, *p* < 0.01) and coronal area (*r* = 0.722, *p* < 0.01), while moderate predictors included axial length (*r* = 0.523, *p* < 0.01), axial width (*r* = 0.521, *p* < 0.01), coronal length (*r* = 0.450, *p* < 0.01), and coronal width (*r* = 0.649, *p* < 0.01).

**Conclusion:**

In patients with thyroid eye disease, lacrimal gland volume can be estimated using axial and coronal areas, which is simpler and more time efficient than calculating volumes.

**Supplementary Information:**

The online version contains supplementary material available at 10.1007/s10792-024-02991-4.

## Introduction

Thyroid eye disease (TED) is an autoimmune disorder of the retrobulbar tissue with a prevalence of 40% in Graves’ disease patients [1]. Lacrimal gland enlargement is a common feature of TED [2–9], and may present clinically with ocular surface discomfort due to increased inflammatory cytokines [3], and reduced tear secretion due to TED associated dacryoadenitis [10]. Lacrimal gland enlargement may serve as a marker of more severe disease and has been positively correlated with the clinical activity score [5, 6, 9].

Lacrimal gland volume is the preferred measurement for assessing the lacrimal gland as it provides a three-dimensional view of the gland and reflects its overall size and shape. However, the process of calculating lacrimal gland volume is not readily clinically translatable due to the expertise, time and advanced software required [11]. Other simpler estimates of lacrimal gland size include the lacrimal gland length and width or area.

Previous studies have shown that simple dimensions can be used to estimate the volume of recti muscles [11, 12]. In a study that included 47 eyes of 47 patients with TED and 47 healthy controls, maximum medial rectus diameter was statistically associated with medial rectus volume (*r* = 0.978, *p* < 0.01) [13]. No previous studies exist assessing the correlations between lacrimal gland dimensions and lacrimal gland volume. The aim of our study is to determine whether the lacrimal gland volume in patients with TED can be estimated using lacrimal gland linear dimensions and area measurements. Additionally, the lacrimal gland prolapse and proptosis values will be calculated, and all measurements will be correlated with age and sex.

## Methods

A retrospective review of MRI scans was conducted on 102 orbits from 51 patients who had TED in Adelaide, South Australia. The study was approved by the Central Adelaide Local Health Network ethics committee and adhered to the principles of the Declaration of Helsinki. All scans were produced using a high field (3-Tesla, 3 T) fat-suppressed contrast-enhanced T1-weighted MRI of the orbits. Contrast-enhanced images were obtained following intravenous administration of standard weight-based dose of gadolinium. Exclusion criteria included age < 18 years and poor image quality. Scans required both axial and coronal views. Data were collected for age and gender.

Patients were evaluated using a Magnetom 3T Skyra scanner (Siemens, Germany) with a conventional turbo spin-echo sequence (TR/TE, 500/15; field of view, 200 × 200 mm; matrix, 512 × 512; slice thickness, 2–3 mm). All patients’ scans were standardised, with axial sections obtained parallel to the optic nerve, and coronal sections positioned perpendicular to the axial plane. The palpebral and orbital lobes were treated as one structure. All measurements were performed using DICOM imaging viewer (OsiriX; Geneva, Switzerland).

### Calculation of lacrimal gland dimensions

**Fig. 1:**
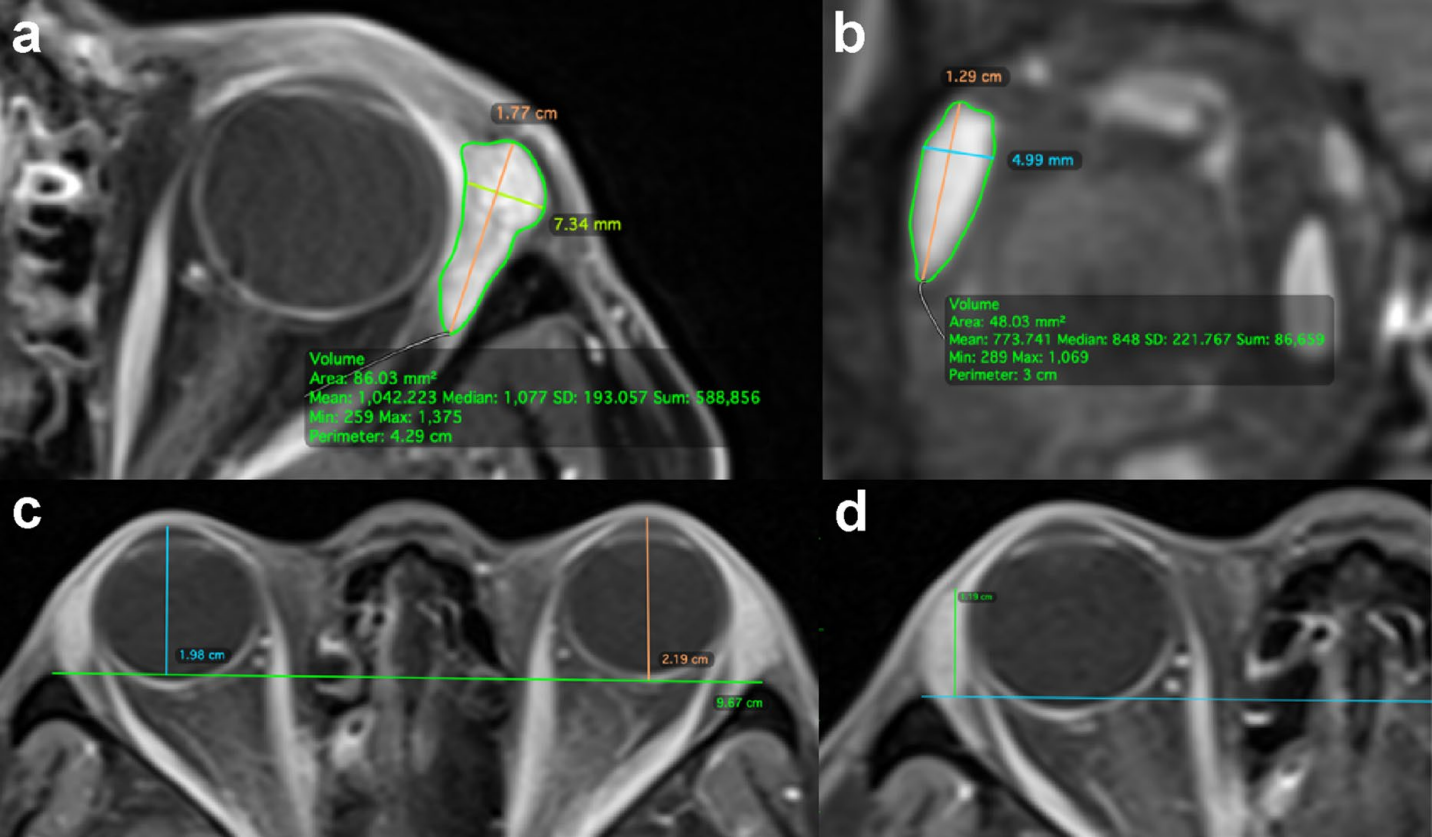
Fat-suppressed contrast-enhanced T1-weighted MRI demonstrating the lacrimal gland quantitative measurements, including, axial length (orange), width (yellow), and area (green) (**a**), coronal length (orange), width (blue), and area (green) (**b**), proptosis value (blue/orange) (**c**), and lacrimal gland prolapse value (green) (**d**)

In both the axial and coronal planes, the image in which the lacrimal gland was most apparent was selected. All measurements were made with the linear length tool. The axial length (AL) was measured from the most-posterior tip to the most anterior-tip of the gland. The axial width (AW) was measured from the lateral edge to the medial edge of the gland at its widest location, perpendicular to the length (Fig. 1a). The coronal length (CL) was measured from the superior tip to the inferior tip of the gland. The coronal width (CW) was measured from the lateral edge to the medial edge of the lacrimal gland at its widest point, perpendicular to the length (Fig. 1b).

### Calculation of proptosis and lacrimal gland prolapse value

On axial scans, multiplanar reconstruction was performed to align the orbits. An interzygomatic line was drawn between the anterior tips of both zygomatic bones. At the midorbit, the proptosis values were calculated as the vertical distance from the interzygomatic line to the anterior cornea (Fig. 1c). At the level of maximum delineated lacrimal gland, the lacrimal gland prolapse (LGP) value was calculated as the vertical distance from the interzygomatic line to the most anterior part of the lacrimal gland (Fig. 1d).

### Calculation of maximum axial and coronal areas

The area of the lacrimal gland was measured using the same slices used for axial and coronal dimensions (Fig. 1a and b).

### Calculation of lacrimal gland volume

The volume was calculated using all consecutive axial slices in which the lacrimal gland was present. The lacrimal gland was manually segmented using the closed polygon tool. The volume was calculated using the region of interest (ROI) volume calculator on the OsiriX software, which works by multiplying area and slice thickness and then adding up individual slice volumes.

### Intrarater reliability

The coefficient of variation (CV) was used to evaluate the intrarater reliability. One author blindly repeated the calculation of the lacrimal gland quantitative measurements in six orbits three weeks after the initial measurement.

### Interrater reliability

This was assessed by two authors using the methods described by Tamboli et al. [14]. The two observers performed lacrimal gland quantitative measurements on the same ten randomly selected MRI scans, blinded to each other’s results. After assessing interrater reliability, one author continued with the remaining scans. Intraclass correlation coefficient (ICC) was used to evaluate the interrater reliability of the two authors.

### Statistics

All statistical analyses were performed using IBM SPSS Statistics for MacOS, Version 27.0 (IBM Corp., Armonk, NY). The level of statistical significance was set at a *p* value of less than 0.05. The following ICC interpretation was used: poor (< 0.50), moderate (0.50–0.75), good (0.75–0.90) and excellent (> 0.90). Independent sample *t*-test was used to evaluate the differences between males and females, as well as right and left orbits. Pearson’s correlation coefficient was used to assess the correlation between each of the quantitative measurements. The following correlation interpretation was used: negligible (0.00–0.10), weak (0.10–0.39), moderate (0.40–0.69), strong (0.70–0.89) and very strong (0.90–1.00) [15]. Multiple linear regression with stepwise regression was used to build a multivariate model that assesses whether certain combinations of quantitative measurements could significantly predict volume. All quantitative measurements were used as predictors for volume in the model.

## Results

One hundred two orbits from 51 patients (34 females and 17 males) were included. The mean age of participants was 59 ± 16 years (18–87 years). There was no significant difference in the mean ages between genders (*p* = 0.724). There was excellent intraclass correlation with CV values being less than 10% for all values except for two. There was excellent interrater reliability. The ICC values for AL, AW, CL, CW, proptosis value, LGP value, axial area, coronal area, and volume for the two observers were 0.93, 0.93, 0.98, 0.85, 0.90, 0.82, 0.81, 0.88, and 0.99, respectively.

The mean values ± standard deviations of lacrimal gland quantitative measurements for all participants, as well as male and female groups, are given in Table 1. No significant difference was found in any lacrimal gland measurement between male and female groups.

**Table 1:** Quantitative measurements of lacrimal glands (mean ± SD) in patients with thyroid eye disease and comparison of male and female sex by the independent sample *t*-test

Measurement	Total (*n* = 102)	Male (*n* = 34)	Female (*n* = 68)	*p*-value
AL (mm)	16.6 ± 3.0	16.2 ± 2.9	16.8 ± 3.1	0.308
AW (mm)	5.9 ± 1.3	5.7 ± 1.4	5.9 ± 1.3	0.391
CL (mm)	16.5 ± 3.7	16.0 ± 3.6	16.7 ± 3.8	0.388
CW (mm)	5.3 ± 1.2	5.0 ± 0.9	5.5 ± 1.3	0.053
Proptosis value (mm)	21.5 ± 3.7	22.1 ± 4.0	21.2 ± 3.6	0.255
LGP value (mm)	11.0 ± 2.9	10.7 ± 3.0	11.1 ± 2.9	0.539
Axial area (mm^2^)	70.3 ± 24.0	65.7 ± 14.5	72.5 ± 27.3	0.175
Coronal area (mm^2^)	67.9 ± 24.2	63.5 ± 24.0	70.1 ± 24.3	0.198
Volume (mm^3^)	933.8 ± 358.5	875.7 ± 343.1	962.9 ± 364.9	0.249

There was no significant difference between the right and left orbits for all lacrimal gland quantitative measurements. A significant negative correlation was found between age and the axial width (*r* = − 0.30, *p* < 0.01), coronal width (*r* = − 0.28, *p* < 0.01), axial area (*r* = − 0.28, *p* < 0.01), coronal area (*r* = − 0.20, *p* < 0.05) and volume (*r* = − 0.28, *p* = 0.05) (Table 2).

**Table 2:** Association between age and lacrimal gland quantitative measurements assessed with Pearson’s correlation coefficient

Measurement	Pearson’s, *r*	*p*-value
AL	− 0.027	0.785
AW	− 0.304	0.002
CL	0.037	0.712
CW	− 0.280	0.004
Proptosis value	0.007	0.946
LGP value	0.026	0.792
Axial area	− 0.281	0.004
Coronal area	− 0.197	0.048
Volume	− 0.278	0.050

With univariate analyses, axial area (*r* = 0.704, *p* < 0.01) and coronal area (*r* = 0.722, *p* < 0.01) were strongly correlated with volume (Table 3). The simple dimensions AL, AW, CL, and CW showed moderate correlation with volume (Table 3). Proptosis value and LGP value were strongly correlated with each other (*r* = 0.736, *p* < 0.01) (Online Resource 1).

**Table 3:** Pearson’s correlation coefficients (*r*) for correlation between lacrimal gland quantitative measurements and volume

Measurement		AL	AW	CL	CW	Proptosis value	LGP value	Axial area	Coronal area
Volume	*r*	0.523	0.521	0.450	0.649	− 0.053	0.094	0.704	0.722
	*p*-value	< 0.01	< 0.01	< 0.01	< 0.01	0.594	0.348	< 0.01	< 0.01

A multiple linear regression model showed strong correlation of combined axial and coronal areas with volume (*R* = 0.843). The model was statistically significant (*R*^2^ = 0.711, *F* (2.99) = 121.752, *p* < 0.01).

## Discussion

Lacrimal gland enlargement is common in TED and may be the initial presenting sign [16]. Lacrimal gland involvement has been correlated with disease severity and ocular surface symptoms [5–7, 9]. Although lacrimal gland volume is the preferred method of assessment, calculating lacrimal gland volumes is not currently feasible due to the additional time and software required. To the best of our knowledge, this is the first study to demonstrate that lacrimal gland areas can be used to estimate lacrimal gland volumes.

Lacrimal gland area measurements can be performed on most existing clinical workspaces, thus negating the need for additional software. The calculation of lacrimal gland area is much more time-efficient than calculating volumes.

Magnetic resonance imaging is the preferred imaging modality for the evaluation of the lacrimal glands because of its superior soft tissue contrast and spatial resolution compared to CT [17]. In particular, fat suppressed contrast-enhanced T1-weighted MRI is most commonly used for evaluating inflammatory or neoplastic disease of the lacrimal gland. This sequence also allows for easier delineation of the lacrimal gland (high intensity) from the surrounding fat (low intensity). The majority of previous studies on the lacrimal gland are based on CT [2–4, 6, 14, 18–20], which offers inferior soft tissue resolution. The superior soft tissue resolution of MRI increases the reproducibility of results, demonstrated by our excellent intrarater and interrater reliability. Comparatively, the ICC values obtained from CT-based studies of the lacrimal gland have been lower, with Bingham et al. [21] reporting an ICC of 0.727 for volume measurements and Bulbul et al. [19] reporting an ICC of less than 0.760 for simple dimensions and volume measurements.

The lacrimal gland volume and area measurements in our study are similar to some previous studies [3, 8], and are positioned within the range of results from other studies [5, 6, 22]. Differences in measurements between papers can be attributed to differences in methods, including imaging modality and sequence used, statistical software, population group and disease activity of participants (e.g. active versus inactive TED).

Our results found that increasing age may be associated with a decrease in lacrimal gland volume, in line with previous studies [5, 23], with Huh et al. reporting that the lacrimal gland volume in control patients was 640 mm^3^ for patients aged 30 and 540 mm^3^ for patients aged 75 [4]. This may be due to increased tissue fibrosis [24]. With time, patients with TED are likely to transition from an active phase into an inactive phase, which is associated with fibrosis and atrophy [25]. Additionally, lacrimal gland volume may decrease as a normal response to age itself [14, 18, 19, 21]. Therefore, when making comparisons of lacrimal gland size, it is important to consider the age of the cohort.

We reported no significant differences between males and females, or between right and left orbits, in all quantitative measurements of the lacrimal glands. This is in concordance with previous studies that measured lacrimal gland parameters in patients with TED [2–5].

Limitations to our study include its retrospective nature and single Australian cohort used. Future prospective follow-up studies may be required to determine if these calculated correlations are maintained during TED therapy, as treatment is likely to cause changes in the lacrimal gland shape.

## Conclusion

In conclusion, our results demonstrate that in patients with TED, the volume of the lacrimal gland has strong correlation with axial and coronal areas, either combined or separately, and moderate correlation with simple dimensions. Therefore, clinicians may use axial and coronal areas to estimate lacrimal gland volume.

## Supplementary Information

Below is the link to the electronic supplementary material.

Supplementary file1 (DOCX 18 KB)

## References

[1] Chin YH, Ng CH, Lee MH, Koh JWH, Kiew J, Yang SP, Sundar G, Khoo CM (2020) Prevalence of thyroid eye disease in Graves’ disease: a meta-analysis and systematic review. Clin Endocrinol (Oxf) 93(4):363–374. 10.1111/cen.14296 32691849 10.1111/cen.14296

[2] Harris MA, Realini T, Hogg JP, Sivak-Callcott JA (2012) CT dimensions of the lacrimal gland in Graves orbitopathy. Ophthalmic Plast Reconstr Surg 28(1):69–72. 10.1097/IOP.0b013e31823c4a3a 22262292 10.1097/IOP.0b013e31823c4a3a

[3] Bingham CM, Harris MA, Realini T, Nguyen J, Hogg JP, Sivak-Callcott JA (2014) Calculated computed tomography volumes of lacrimal glands and comparison to clinical findings in patients with thyroid eye disease. Ophthalmic Plast Reconstr Surg 30(2):116–118. 10.1097/iop.0000000000000015 24448234 10.1097/IOP.0000000000000015

[4] Huh HD, Kim JH, Kim SJ, Yoo JM, Seo SW (2016) The change of lacrimal gland volume in Korean patients with thyroid-associated ophthalmopathy. Korean J Ophthalmol 30(5):319–325. 10.3341/kjo.2016.30.5.319 27729751 10.3341/kjo.2016.30.5.319 PMC5057007

[5] Huang D, Luo Q, Yang H, Mao Y (2014) Changes of lacrimal gland and tear inflammatory cytokines in thyroid-associated ophthalmopathy. Invest Ophthalmol Vis Sci 55(8):4935–4943. 10.1167/iovs.13-13704 24994866 10.1167/iovs.13-13704

[6] Byun JS, Moon NJ, Lee JK (2017) Quantitative analysis of orbital soft tissues on computed tomography to assess the activity of thyroid-associated orbitopathy. Graefes Arch Clin Exp Ophthalmol 255(2):413–420. 10.1007/s00417-016-3538-0 27838736 10.1007/s00417-016-3538-0

[7] Jiang C, Li X, Zhao M, Dend H, Huang J, Liu D, Xu X (2019) Efficacy of 99mTc-DTPA orbital SPECT/CT on the evaluation of lacrimal gland inflammation in patients with thyroid associated ophthalmopathy. Zhong Nan Da Xue Xue Bao Yi Xue Ban 44(3):322–328. 10.11817/j.issn.1672-7347.2019.03.014 30971526 10.11817/j.issn.1672-7347.2019.03.014

[8] Hu H, Xu XQ, Wu FY, Chen HH, Su GY, Shen J, Hong XN, Shi HB (2016) Diagnosis and stage of Graves’ ophthalmopathy: efficacy of quantitative measurements of the lacrimal gland based on 3-T magnetic resonance imaging. Exp Ther Med 12(2):725–729. 10.3892/etm.2016.3389 27446267 10.3892/etm.2016.3389 PMC4950689

[9] Zhao RX, Shi TT, Luo S, Liu YF, Xin Z, Yang JK (2022) The value of SPECT/CT imaging of lacrimal glands as a means of assessing the activity of Graves’ orbitopathy. Endocr Connect. 10.1530/ec-21-0590 35015696 10.1530/EC-21-0590 PMC8859942

[10] Allam IY, Lazreg S, Shafik Shaheen M, Doheim MF, Mohammed MA (2021) Ocular surface changes in patients with thyroid eye disease: an observational clinical study. Clin Ophthalmol 15:2481–2488. 10.2147/opth.S317708 34163131 10.2147/OPTH.S317708 PMC8214558

[11] Szucs-Farkas Z, Toth J, Balazs E, Galuska L, Burman KD, Karanyi Z, Leovey A, Nagy EV (2002) Using morphologic parameters of extraocular muscles for diagnosis and follow-up of Graves’ ophthalmopathy: diameters, areas, or volumes? AJR Am J Roentgenol 179(4):1005–1010. 10.2214/ajr.179.4.1791005 12239055 10.2214/ajr.179.4.1791005

[12] Weis E, Heran MK, Jhamb A, Chan AK, Chiu JP, Hurley MC, Rootman J (2012) Quantitative computed tomographic predictors of compressive optic neuropathy in patients with thyroid orbitopathy: a volumetric analysis. Ophthalmology 119(10):2174–2178. 10.1016/j.ophtha.2012.04.021 22709420 10.1016/j.ophtha.2012.04.021

[13] Bontzos G, Papadaki E, Mazonakis M, Maris TG, Tsakalis NG, Drakonaki EE, Detorakis ET (2022) Extraocular muscle volumetry for assessment of thyroid eye disease. J Neuroophthalmol 42(1):e274–e280. 10.1097/wno.0000000000001339 34629402 10.1097/WNO.0000000000001339

[14] Tamboli DA, Harris MA, Hogg JP, Realini T, Sivak-Callcott JA (2011) Computed tomography dimensions of the lacrimal gland in normal Caucasian orbits. Ophthalmic Plast Reconstr Surg 27(6):453–456. 10.1097/IOP.0b013e31821e9f5d 21659915 10.1097/IOP.0b013e31821e9f5d

[15] Schober P, Boer C, Schwarte LA (2018) Correlation coefficients: appropriate use and interpretation. Anesth Analg 126(5):1763–1768. 10.1213/ane.0000000000002864 29481436 10.1213/ANE.0000000000002864

[16] Khu J, Freedman KA (2017) Lacrimal gland enlargement as an early clinical or radiological sign in thyroid orbitopathy. Am J Ophthalmol Case Rep 5:1–3. 10.1016/j.ajoc.2016.10.005 29503935 10.1016/j.ajoc.2016.10.005 PMC5757804

[17] Ferreira TA, Saraiva P, Genders SW, Buchem MV, Luyten GPM, Beenakker JW (2018) CT and MR imaging of orbital inflammation. Neuroradiology 60(12):1253–1266. 10.1007/s00234-018-2103-4 30310941 10.1007/s00234-018-2103-4 PMC6244997

[18] Lee JS, Lee H, Kim JW, Chang M, Park M, Baek S (2013) Computed tomographic dimensions of the lacrimal gland in healthy orbits. J Craniofac Surg 24(3):712–715. 10.1097/SCS.0b013e31827fecc0 23715005 10.1097/SCS.0b013e31827fecc0

[19] Bulbul E, Yazici A, Yanik B, Yazici H, Demirpolat G (2016) Evaluation of lacrimal gland dimensions and volume in Turkish population with computed tomography. J Clin Diagn Res 10(2):Tc06-8. 10.7860/jcdr/2016/16331.7207 27042554 10.7860/JCDR/2016/16331.7207 PMC4800620

[20] Nawaz S, Lal S, Butt R, Ali M, Shahani B, Dadlani A (2020) Computed tomography evaluation of normal lacrimal gland dimensions in the adult Pakistani population. Cureus 12(3):e7393. 10.7759/cureus.7393 32337120 10.7759/cureus.7393 PMC7179983

[21] Bingham CM, Castro A, Realini T, Nguyen J, Hogg JP, Sivak-Callcott JA (2013) Calculated CT volumes of lacrimal glands in normal Caucasian orbits. Ophthalmic Plast Reconstr Surg 29(3):157–159. 10.1097/IOP.0b013e3182859751 23503056 10.1097/IOP.0b013e3182859751

[22] Ugradar S, Zimmerman E, Parunakian E, Kang J, Cockerham K, Douglas RS (2023) Change in lacrimal gland volume and aqueous tear production following treatment with teprotumumab. Clin Exp Ophthalmol. 10.1111/ceo.14208 36723406 10.1111/ceo.14208

[23] Rana K, Juniat V, Patel S, Selva D (2022) Normative lacrimal gland dimensions by magnetic resonance imaging in an Australian cohort. Orbit. 10.1080/01676830.2022.2055085 35470758 10.1080/01676830.2022.2055085

[24] Obata H, Yamamoto S, Horiuchi H, Machinami R (1995) Histopathologic study of human lacrimal gland. Statistical analysis with special reference to aging. Ophthalmology 102(4):678–686. 10.1016/s0161-6420(95)30971-2 7724184 10.1016/s0161-6420(95)30971-2

[25] Bothun ED, Scheurer RA, Harrison AR, Lee MS (2009) Update on thyroid eye disease and management. Clin Ophthalmol 3:543–551. 10.2147/opth.s5228 19898626 10.2147/opth.s5228 PMC2770865

