# Supplementary material for: Radiological measurements of lacrimal gland in thyroid eye disease
Source: Int Ophthalmol. 2024 Feb 6;44(1):11. doi: 10.1007/s10792-024-02991-4 (PMC10847191; doi:10.1007/s10792-024-02991-4)
Supplement: Supplementary file 1 — Supplementary file1 (DOCX 18 KB) [file 10792_2024_2991_MOESM1_ESM.docx]

| **Online Resource 1. Pearson's correlation coefficients (r) for quantitative measurements of the lacrimal gland** | | | | | | | | | | |
| --- | --- | --- | --- | --- | --- | --- | --- | --- | --- | --- |
| **Measurement** | | AL | AW | CL | CW | Proptosis | LGP | Axial Area | Coronal Area |  |
| AW | r | 0.210 |  |  |  |  |  |  |  |  |
|  | p-value | 0.034 |  |  |  |  |  |  |  |  |
| CL | r | 0.101 | 0.209 |  |  |  |  |  |  |  |
|  | p-value | 0.313 | 0.035 |  |  |  |  |  |  |  |
| CW | r | 0.340 | 0.509 | 0.371 |  |  |  |  |  |  |
|  | p-value | <0.01 | <0.01 | <0.01 |  |  |  |  |  |  |
| Proptosis | r | 0.067 | 0.114 | -0.269 | 0.043 |  |  |  |  |  |
|  | p-value | 0.503 | 0.254 | <0.01 | 0.669 |  |  |  |  |  |
| LGP | r | 0.206 | 0.082 | -0.195 | 0.007 | 0.736 |  |  |  |  |
|  | p-value | 0.038 | 0.413 | 0.049 | 0.946 | <0.01 |  |  |  |  |
| Axial Area | r | 0.734 | 0.592 | 0.118 | 0.479 | 0.148 | 0.229 |  |  |  |
|  | p-value | <0.01 | <0.01 | 0.239 | <0.01 | 0.139 | 0.021 |  |  |  |
| Coronal Area | r | 0.282 | 0.525 | 0.743 | 0.760 | -0.104 | -0.084 | 0.431 |  |  |
|  | p-value | 0.004 | <0.01 | <0.01 | <0.01 | 0.300 | 0.399 | <0.01 |  |  |
| Volume | r | 0.523 | 0.521 | 0.450 | 0.649 | -0.053 | 0.094 | 0.704 | 0.722 |  |
|  | p-value | <0.01 | <0.01 | <0.01 | <0.01 | 0.594 | 0.348 | <0.01 | <0.01 |  |
